# Hydrological Modelling for Siberian Crane *Grus Leucogeranus* Stopover Sites in Northeast China

**DOI:** 10.1371/journal.pone.0122687

**Published:** 2015-04-15

**Authors:** Haibo Jiang, Chunguang He, Lianxi Sheng, Zhanhui Tang, Yang Wen, Tingting Yan, Changlin Zou

**Affiliations:** 1 State Environmental Protection Key Laboratory of Wetland Ecology and Vegetation Restoration, School of Environment, Northeast Normal University, Changchun, China; 2 Momoge National Nature Reserve of Jilin, Zhenlai, China; Shandong University, CHINA

## Abstract

Habitat loss is one of the key factors underlying the decline of many waterbird species, including Siberian Crane (*Grus leucogeranus*), a threatened species worldwide. Wetlands are the primary stopover for many waterbirds and restoration of these wetlands involves both hydrological restoration and water resource management. To protect the stopover sites of Siberian Cranes, we collected Siberian Crane stopover numbers, meteorological and hydrological data, and remote sensing data from 2008 to 2011 in Momoge National Nature Reserve, one of the largest wetlands in northeastern China. A model was developed to estimate the suitability of *Siberian Crane stopover* sites. According to our results, the most suitable daily water level for Siberian Cranes between 2008 and 2012 occurred in the spring of 2008 and in the *Scirpus planiculmis* growing season and autumn of 2010. We suggest a season-dependent water management strategy in order to provide suitable conditions at Siberian Crane stopover sites.

## Introduction

Natural wetlands have become increasingly scarce worldwide due to drainage and agricultural development projects [1, 2]. Loss of habitat is the most important factor underlying the decline of many waterbird species around world [3–6]. As a result, appropriate ecosystem management has been initiated to protect and restore wetland habitat in both interior and coastal regions [7–11]. To date, most research has been focused on vegetation restoration [12] as well as the associated responses of animal populations [13], especially birds [14–21]. Currently, water management strategies based on the relationship between waterbirds and wetland water level are the main method for protecting waterbirds [22, 23]. However, the most effective strategies for the management of water resources for many large—waders—during stopover remain poorly understood.

Siberian Crane (*Grus leucogeranus*) is listed as a threatened species according to the IUCN “red list” [24]. The global population of Siberian Cranes is estimated to be approximately 4000 individuals, which are separated into three recognized populations (i.e., eastern, central, and western) based on migration routes. The eastern population is the largest, accounting for approximately 99% of the total number [25]. In autumn, Siberian Cranes migrate from Northeast Siberia in Russia to Poyang Lake in the Yangtze River basin of China, and return to Siberia the following spring [26]. During this migration, which can exceed *5000 km*, the Siberian Cranes stop for 1–2 months to replenish their energy at stopover sites, such as Momoge Nature Reserve and Zhalong Nature Reserve, located in the West Songnen Plain, China. Previous ecological studies have concluded that there are two basic requirements for Siberian Cranes at stopover sites, suitable daily water level between 0 and 50 cm, and adequate food resources [27–30]. The primary food for Siberian Cranes at stopover sites is the tuber of *Scirpus planiculmis* [31], one an aquatic plant with a high reproduction capacity in areas with a daily water level between 0 and 30 cm [32]. Therefore, several locations within the flood plains of the Nenjiang, Tao’er, and Huolin Rivers have been selected as the primary sites for Siberian Cranes during migration. Because of the flat terrain, these flood plains form numerous small lakes and marshes, which serve as ideal stopover sites for Siberian Cranes.

In the past six decades, many wetlands have dried or been reclaimed because of changes in land use and climate, thus detrimentally affecting waterbird habitat [33]. With the loss of many wetland stopover sites, the migration process of the Siberian Crane has been seriously threatened. The government of Jilin Province enacted a water diversion project in 2008, intended to protect stopover sites and agricultural development, which illustrated how the distribution of water resources had become an important issue.

Currently, the study of the protection of keystone species based on accurate hydrological simulation is limited. In this study, the relationship between the number of Siberian Cranes and the hydrological conditions at stopover sites was examined. The objectives of our study include the following: (1) to estimate the changes in daily water level; (2) to determine the suitable daily water level changes and water supplementation for Siberian Cranes; and (3) to develop an effective strategy for the protection of wetlands and Siberian Cranes under a multiple-use water supply system.

## Methods

### Ethics statement

Our research was conducted in strict accordance with animal care permits issued by China’s State Forestry Administration. It was conducted on public land with permission from the local government and residents. No further specific permissions were required. The field work did not involve endangered or protected species and did not require permits from an Institutional Animal Care and Use Committee (IACUC) or an equivalent animal ethics committee because there was no physical handling or other potentially detrimental activity toward the animals. In all of our field work, care was taken to minimize any negative impact on the welfare of the animals involved, including the selection of appropriate methods for monitoring the Siberian Cranes and surveying habitat.

### Study area

Momoge National Nature Reserve (45°42′25″-46°18′0″E, 123°27′0″-124°4′33″N) is located in Zhenlai County of Jilin Province in China and covers an area of 1440 km^2^ [34]. It is one of the largest wetlands in northeast China, situated in a semiarid area with a continental monsoon climate of the North Temperate Zone [35]. The annual precipitation, evapotranspiration and mean temperature from 1950 to 2012 were 385.69 mm, 1530.13 mm and 4.97°C, respectively.

**Fig 1:**
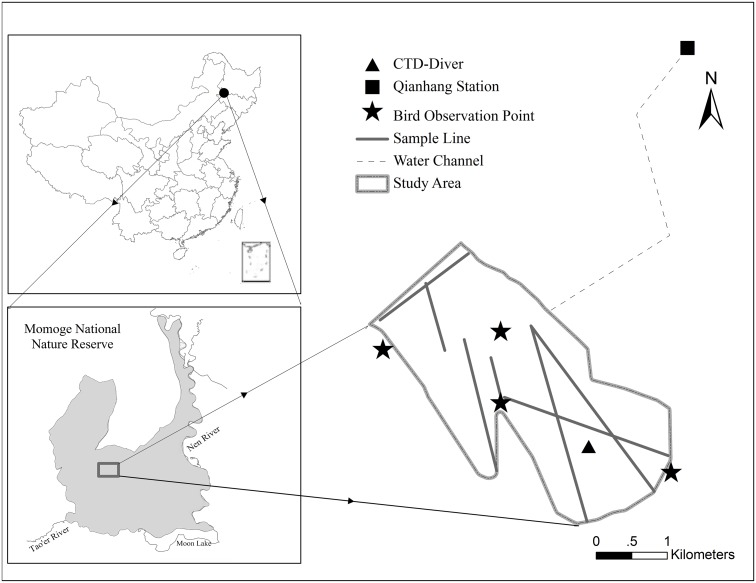
Location of the study area and bird observation points, distribution of Qianhang station and sample lines.

The study area, Etou wetland, has an area of 8.1 km^2^ and is located centrally within the nature reserve (Fig 1). Qianhang Station, 5 km to the northeast of study area, was established to transfer water from the Nenjiang River for irrigation and to collect flow in paddy fields. In recent years, water diversion has begun in June, the farming season in the Songnen Plain. There are four pumps in Qianhang Station, with a flowrate of 9000 m^3^/h per pump. Since 2008, the nature reserve has purchased water from Qianhang Station to maintain suitable waterbird habitat in the Etou wetland. In the spring of 2012, approximately 3800 Siberian Cranes, accounting for 95% of the world population, used Etou wetland as a stopover site, making it one of the most important stopover sites in the world for this species.

### Data collection

#### Siberian Crane data

Siberian Cranes stop at Momoge National Nature Reserve from the beginning of April to the middle of May in spring and from the middle of September to the beginning of November in autumn each year. The number of Siberian Cranes was used as an evaluation index of wetland conditions. Because the number of Siberian Cranes might be affected by various migration-related factors, the data obtained on a given day could not be used for analysis. Instead, the cumulative stopover number obtained in four different locations was used as an evaluation index, which was recorded every two days during the stay of Siberian Cranes. The observation time was from 8 am to 10 am, the peak time of the Siberian Crane’s feeding. All data were taken from the nature reserve in spring and autumn from 2008 to 2010 and in the spring of 2011 (S1 File).

#### Meteorological and hydrological data

Meteorological data for Zhenlai County were obtained from the China Meteorological Administration (http://www.cma.gov.cn/), including daily precipitation, air pressure, wind speed, etc. from 2001 to 2012. Daily evapotranspiration can be calculated using the following equation [36]:

E=Δe(0.001+0.232W) (1)

Where *E* is daily evapotranspiration (mm), Δ*e* is daily difference of saturation vapor pressure (Pa), and *W* is daily wind speed (m/s).

The daily water supplementation data were obtained from Qianhang Station from 2008 to 2012. The CTD-Diver water level logger (Dutch Eijkelkamp Company) was also used to measure water levels every one hour in 2012 (S2 File). All data were expressed as daily average values. Seven lines in the study area were established in May 2012 for the measurement of water levels (Fig 1, S3 File).

Remote sensing data. Landsat Thematic Mapper (TM) and Landsat Earth Thematic Mapper (ETM) remote sensing images were obtained from the International Scientific Data Service Platform (http://datamiror.Csdb.cn/index.jsp). The resolution of Landsat TM/ETM images is 30 m. The collection dates for these data were 2008.8.5 (ETM), 2009.6.13 (TM), 2009.8.16 (TM), 2010.7.2 (TM), 2010.8.11 (ETM), and 2011.7.29 (ETM). Strips of ETM images were repaired using ENVI 4.7, and then all remote images were calibrated. The resulting data were geo-referenced to the same coordinates using GPS information of feature points (road intersections etc., with a total of five points) and a second-order polynomial transformation with nearest-neighbor resampling using ArcGIS 9.3. The error of Geo-referencing was ensured within a pixel.

### Model development

#### Relationship between wetland area and daily water level

In the study area, the source of the majority of water comes from natural rainfall and the water supplied from Qianhang Station. The outflow includes evapotranspiration and infiltration. Because wetlands are composed of lakes with a very limited number of plants and most lands contain saline-alkaline soil, the evapotranspiration from plants was not included in the data analysis. To study the changes in daily water level, Equation (2) was used:

$$H_{n}=\sum_{i=1}^{n}\frac{Q_{i}}{A_{i}}+\sum_{i=1}^{n}R_{i}-\sum_{i=1}^{n}E_{vi}-\sum_{i=1}^{n}F_{i}+h_{0}$$ (2)

Where *H*_*n*_ is the daily water level value on the *n*th day (m), *Q*_*i*_ is the drainage value on the *i*th day (m^3^), *A*_*i*_ is the wetland area on the *i*th day (m^2^), *R*_*i*_ is the precipitation on the *i*th day (m), *E*_*vi*_ is the evapotranspiration on the *i*th day (m), *F*_*i*_ is the infiltration on the *i*th day (m), and *h*_*0*_ is the daily water level before water supplementation (m).

The daily water level change (Equation (3)) caused by water supplementation can be derived from Equation (2):

$$h_{n}=\sum_{i=1}^{n}\frac{Q_{i}}{A_{i}}=H_{n}-\sum_{i=1}^{n}R_{i}+\sum_{i=1}^{n}E_{vi}+\sum_{i=1}^{n}F_{i}-h_{0}$$ (3)

Where *h*_*n*_ is the daily water level change due to water supplementation (m).

Equations (2) and (3) are used to estimate the relationship between wetland area and daily water level change. The daily water level change of 2012 can be estimated based on the wetland area and water inflow. A model was developed to calculate the daily water level. To validate the precision and accuracy, the predictive capability of these equations is evaluated by average error (*AE*), average absolute error (*AAE*), and root-mean squared error (*RMSE*):

$$AE=\sum_{i=1}^{n}\left(y_{i}-{\hat{y}}_{i}\right)/n$$ (4)

$$AAE=\sum_{i=1}^{n}\left|y_{i}-{\hat{y}}_{i}\right|/n$$ (5)

$$RMSE=\sqrt{\sum_{i=1}^{n}{\left(y_{i}-{\hat{y}}_{i}\right)}^{2}/n}$$ (6)

Where *y*_*i*_ is the measured daily water level on the *i*th day (m), ${\hat{y}}_{i}$ is the simulative water level on the *i*th day (m), and *n* is the number of observations.

#### Changes in daily water level

Using the methods described above, we calculated the changes in daily water level from 2008 to 2011, corrected the results according to records. In addition, the wetland area and perimeter were obtained from remote sensing images using ArcGIS 9.3 and then corrected using field measurements. The wetland area and perimeter, along with the remote sensing image, were evaluated using Geo-statistical Analyst in ArcGIS 9.3 based on daily water level distribution of sample lines in 2012 and the trend of changes in daily water level for four years. The difference between calculated and measured results was compared by a paired sample *t* test in SPSS 20.0.

#### Hydrological characteristics

In the study area, the daily water level was constantly changing when Siberian Cranes stopped at the stopover sites. However, only areas with daily water level changes within 0–50 cm are suitable for the Siberian Crane over the entire stopover period. As such, these areas were defined as suitable stopover areas and determined using Geo-statistical Analyst using ArcGIS 9.3. Suitable wetland area, perimeter, patch density index (*P*_*D*_), path splitting index (*F*_*i*_), and fragmentation index (*F*_*l*_), which reflected the hydrological characteristics of stopover sites, were selected to analyze the relationship with the number of Siberian Cranes:

$$P_{D}=\sum n_{i}/A$$ (7)

$$F_{i}=\sqrt{n_{i}/A}/2(A_{i}/A)$$ (8)

$$F_{l}=L_{i}/A_{i}$$ (9)

Where *n*_*i*_ is the number of water surface patches, *A* is the area of research region, *A*_*i*_ is the area of suitable water surfaces, and *L*_*i*_ is the perimeter of suitable water surfaces.

## Results and Discussion

### Relationship between wetland area and daily water level

**Fig 2:**
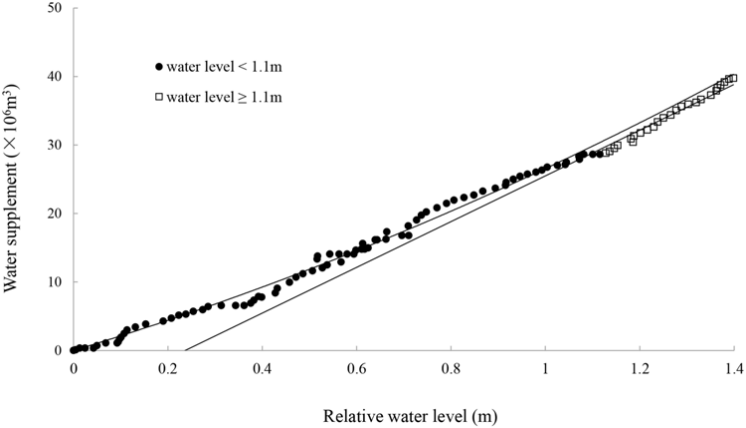
Plot of water supplementation against relative daily water level.

Fig 2 shows the changes in daily water level due to water supply in 2012 calculated by Equation (3). The regression analysis shows a non-linear relationship between water supplementation and daily relative water level (*y* = 5.70*x* ^2^+20.87*x*, *R^2^* = 0.99) when the daily relative water level is less than 1.1 m. When the relative daily water level is greater than 1.1 m, the water supplementation is linearly related to daily relative water level (*y* = 33.39*x*-7.90, *R^2^* = 0.96). Therefore, the water supplementation increases with the increase in the daily water level at the beginning of water diversion. When the water surface reaches the boundary of the road, the daily water level rises vertically, meaning that the water supplementation required to raise the water to the same level daily is approximately constant. This is due to the terrain of the study area, which is low in the middle and high on all sides. In addition, to protect the Siberian Crane and develop tourism, a road at the border of the Etou wetland was built by the nature reserve, leading to terrain with a vertical rise at the boundary of the study area.

The relationship between wetland area and daily water level changes related to water supply (*h*) can be derived from regression equations (Equation (10)):

$$\begin{array}{l} \;A=11392076.19h+20867793.41\;h<1.10\text{m}\\ A=33394168.46\;h\geq 1.10\text{m} \end{array}$$ (10)

**Fig 3:**
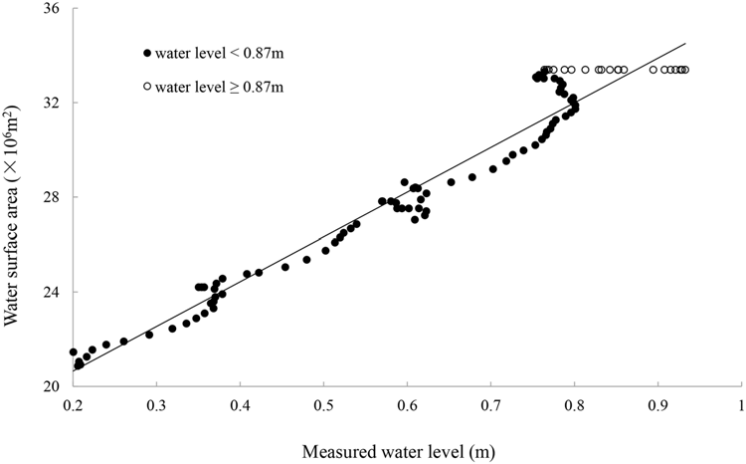
Plot of simulative wetland area against the measured daily water level.

We analyzed the relationship between wetland area calculated from *h* and measured daily water level (*H*). As shown in Equation (11) (*R^2^* = 0.96), we observed a linear relationship between wetland area and *H* when *H* is < 0.87 m, and a constant of *A* when *H* is > 0.87 m (Fig 3).

$$\begin{array}{l} A=18915191.03H+16865095.52\;H<0.87\text{m}\\ A=33394168.46\;H\geq 0.87\text{m} \end{array}$$ (11)

We further compared the results obtained from our model with daily water level measured in 2012. The average error (*AE*), average absolute error (*AAE*), and root-mean squared error (*RMSE*) were 0.02, 0.03, and 0.04, respectively, indicating a high accuracy of our model.

### Daily water level calculation using our model

**Fig 4:**
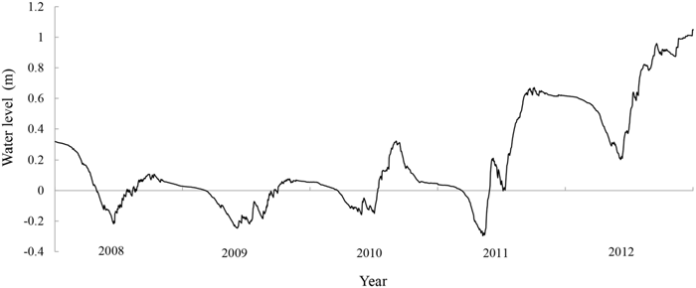
Trend of daily water level changes in the study area from 2008 to 2012.

The changes in daily water level from 2008 to 2011 were calculated using the simulation model in 2012. The paired sample *t* test used to examine the differences in wetland area and perimeter between our estimation and remote sensing interpretation suggests that our model is relatively accurate (*p* = 0.50 for wetland area, *p* = 0.20 for perimeter). The trends of changes in daily water level are consistent from 2008 to 2012 (Fig 4), showing a rapid decline in spring caused by high evapotranspiration, low precipitation and insufficient water supplementation, and then an increase in summer due to rainfall and water supplementation from Qianhang Station and rainfall. After water supplementation stops, daily water level starts to decline in autumn before the wetland ultimately freezes in winter.

As shown in Fig 4, the daily water level reached the minimum in 2009 due to a very limited water supply. The daily water levels in 2008 and 2010 are comparable. In 2011 and 2012, there were sharp rises in the daily water level because of large amounts of water supplementation.

### Distribution of suitable stopover areas

**Fig 5:**
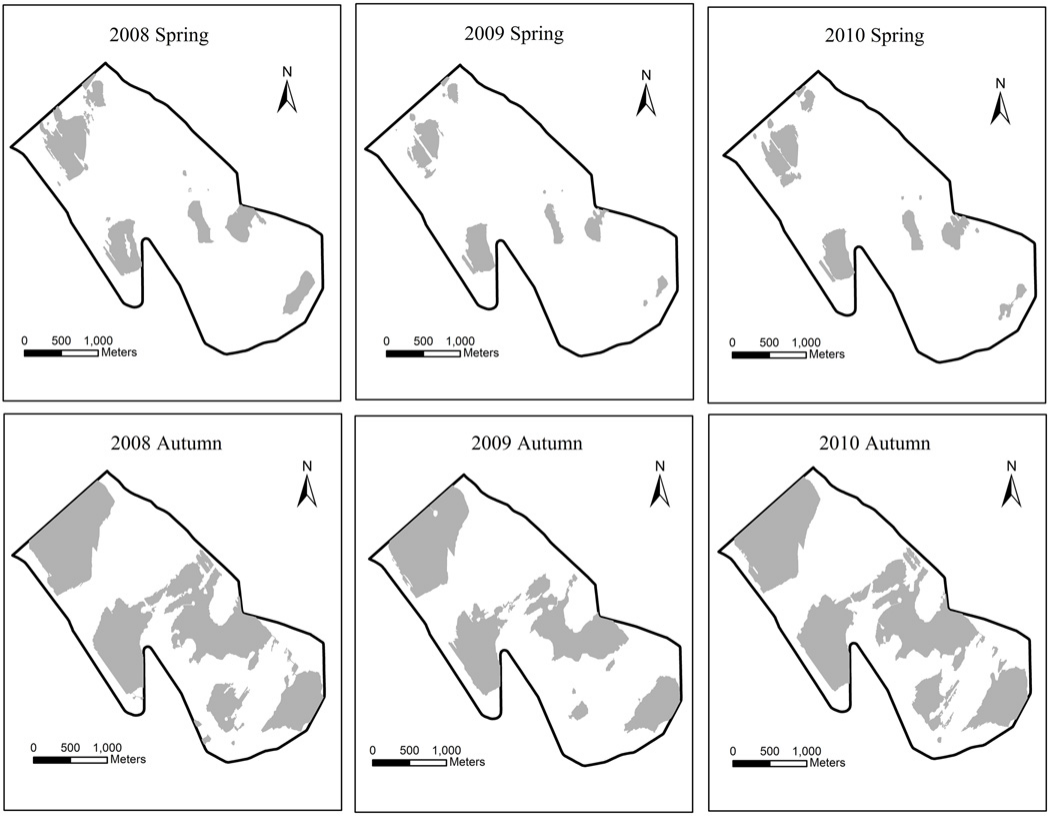
Suitable Siberian Crane stopover areas in spring and autumn from 2008 to 2010.

The distributions of suitable stopover areas in spring and autumn from 2008 to 2010 are shown in Fig 5. Because the daily water level fluctuated greatly during the spring of 2011, there were no suitable stopover areas for the Siberian Crane.

### Relationship between cumulative stopover number and fragmentation indices

Table 1 shows the fragmentation indices of suitable stopover areas in spring and autumn from 2008 to 2010. The cumulative number of Siberian Cranes increased with increasing suitable wetland area and perimeter but decreased with increasing of *P*_*D*_, *F*_*i*_, and *F*_*l*_ (Table 1). These findings suggest that a large suitable wetland area, perimeter and limited fragmentation are important hydrological conditions for stopover *site suitability*. However, the fragmentation index of suitable stopover areas should be interpreted with caution because the fragmentation of suitable stopover areas was different from that of study area.

**Table 1:** Cumulative number of Siberian Cranes during stopover and fragmentation indices of suitable stopover area from 2008 to 2011.

Season	Year	Accumulative number	Area (10^5^m^2^)	Perimeter (10^4^m)	*P*_*D*_ (10^-5^)	*Fi* (10^-3^)	*F*_*1*_ (10^-3^)
Spring	2008	26063	10.57	1.92	1.32	5.04	18.17
	2009	10625	5.98	1.38	2.17	8.58	23.08
	2010	22523	7.81	1.57	1.41	6.04	20.14
	2011	42706	0	0	0	0	0
Autumn	2008	22697	32.56	3.40	0.25	1.24	1.04
	2009	20302	25.31	2.54	0.28	1.49	1.00
	2010	34616	32.76	3.44	0.24	1.23	1.05

Although the cumulative number of Siberian Cranes reached a maximum in the spring of 2011, the conditions of the suitable stopover area were not regarded as optimum due to the sharp fluctuations in the daily water level, which could pose a risk to Siberian Cranes and affect the germination and growth of *Scirpus planiculmis*. However, in the spring of 2008 and autumn of 2010, the daily water level change remained relatively stable with large suitable water area and limited fragmentation. Therefore, we concluded that from 2008 to 2011, the spring of 2008 and autumn of 2010 were the best seasons for Siberian Crane stopover.

It is noteworthy that the selection of a stopover site depends not only on daily water level but also on food resources. To provide enough food resources, the daily water level should be maintained within a suitable range in the growing season of *Scirpus planiculmis*, whose one-year-old tubers are the main food for the Siberian Crane. Therefore, the higher cumulative number in autumn of 2010 and spring of 2011 indicates a suitable daily water level during 2010 for the growth of *Scirpus planiculmis*. In conclusion, from 2008 to 2012, the best daily water level occurred in the spring of 2008, in the *Scirpus planiculmis* growing season, and autumn of 2010.

### Maintenance of suitable daily water level

**Fig 6:**
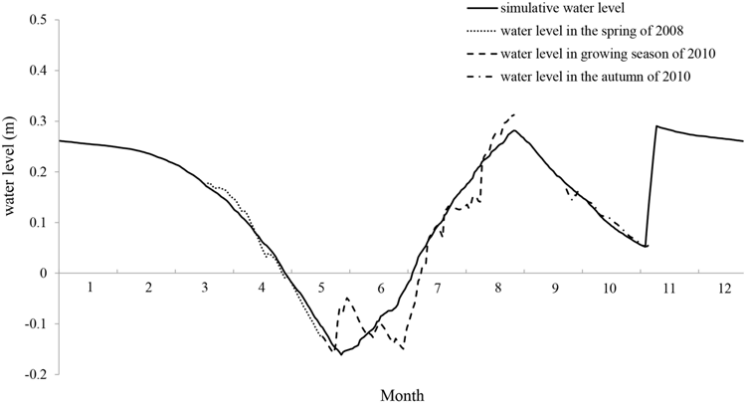
Simulation of suitable daily water level for the study area.

The study area is located in Songnen Plain, a semi-arid region, where management of limited water resources is designed for *multiple-use*. We need to develop an effective water management strategy to balance the ecological and economic benefits and effectively maintain stopover habitat for Siberian Cranes. We estimated the annual daily water level based on changes in daily water level in the spring of 2008, during the *Scirpus planiculmis* growing season, and in autumn of 2010 (Fig 6). In summer, *water should be provided* based on the available resources in Qianhang Station and *adaptability of plants*. To maintain the daily water level in a suitable range for *Scirpus planiculmis* growth, our management plant requires only one pump working for 10 hours/day in June at a daily flowrate of 9×10^4^ m^3^. In July and August, the rainy seasons, only one pump needs to work for 16 hours/day at a daily flowrate of 1.44×10^5^ m^3^ to ensure suitable conditions for the growth of *Scirpus planiculmis* in summer and Siberian Crane stopover in autumn. After Siberian Cranes depart in the middle of November, each pump needs to work for 22.5 hours/day at a daily flowrate of 8.10×10^5^ m^3^ for six days to maintain a suitable daily water level in these areas for the following spring. This plan could ultimately result in the efficient water distribution at the current Siberian Crane stopover sites.

**Fig 7:**
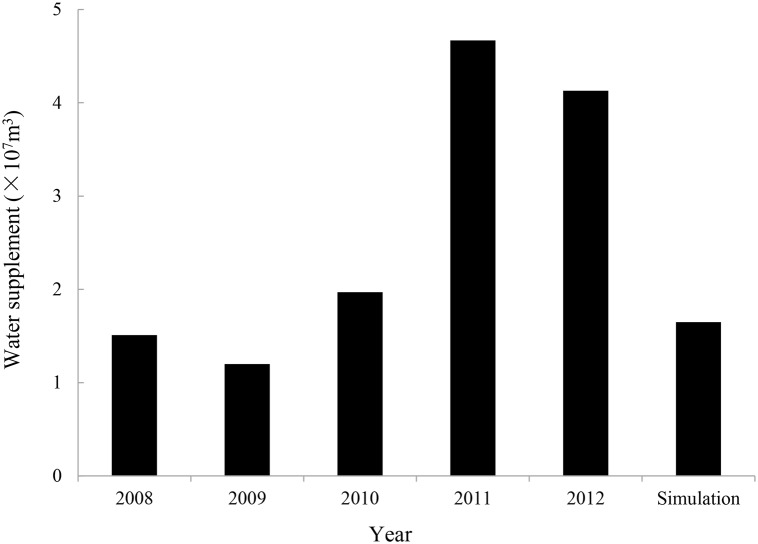
Total water supplementation of the study area from 2008 to 2012 and simulated water supplementation.

Based on our results, an annual water supply of 1.65×10^7^ m^3^ is needed to achieve a suitable daily water level, which is lower than that in 2010 but higher than that in 2008, the two years with relative high numbers Siberian Cranes (Fig 7). Siberian Crane stopover was seriously affected in the autumn of 2011 and 2012 (< 150 individuals), when the water supply was 2-3-fold greater than our estimation. Therefore, excessive water supplementation may greatly affect Siberian Crane stopover in comparison to limited water supplementation. We believe that our model could provide reliable results for daily water level estimation.

It is important to note that the simulation method and water supplementation strategy in this wetland restoration are different from previous studies. Our study area is relatively smaller and was focused on the protection of a keystone species, while previous studies investigated entire reserves or drainage basins [37–39]. In those studies, hydrological control was not accurate and the protection strategy of keystone species was not clear. Thus, our study builds on the previous studies by addressing these questions.

## Conclusions

Hydrological restoration is an effective way to protect Siberian Crane stopover sites. In this study performed in Momoge National Nature Reserve, we demonstrated that daily water level was important to the suitability of Siberian Crane stopover sites. We also developed a model to calculate the suitable daily water level based on data from 2008 to 2011. In addition, an effective strategy to protect Siberian Cranes stopover habitat has been established. The model and strategy to protect Siberian Cranes in this area can be a reference point for restoring large wader stopover sites in Songnen Plain or other areas of the world. More studies are needed before our strategy applied in other contexts.

## Supporting Information

S1 File Siberian Crane data in spring and autumn from 2008 to 2010 and in the spring of 2011. (XLSX)

S2 File Water level data measured by the CTD-Diver water level logger every one hour in 2012. (XLSX)

S3 File Water level data of seven lines measured in the study area in May 2012. (XLSX)
